# Scoliosis from the perspective of a fifteen year old

**DOI:** 10.1186/1748-7161-8-S1-P18

**Published:** 2013-06-03

**Authors:** NC Byskosh

**Affiliations:** 1 Scoliosis Patient, Barrington Hills, IL, USA

## Background

The self-body image and mentality of scoliosis patients, not only the body physically, is affected by scoliosis treatment, including bracing. Through wearing a brace for five years, I look back on my past, and analyze my encounters with physicians, friends, and family, to see which actions they took, or words they spoke, that were comforting, or helpful, and also, examine those of which were not as helpful, to confront physicians and all people affected by scoliosis to express my opinion on how to address scoliosis.

## Aims

To inform physicians of my own perspective on scoliosis, as a scoliosis patient, and answer several questions regarding my experiences. I feel that it is important to discuss my experience as a patient, so physicians come to understand how bracing affects a child physically, emotionally, and socially.

## Methods

As a scoliosis patient, I feel I am able to evaluate and express my opinion of my experiences with having scoliosis. I have worn a brace for five years, four of which included 22 hours a day of wearing the brace. I have travelled to doctors in the United States, Germany, Poland, and Spain, and have also been doing back exercises for years, and spoken to other scoliosis patients.

## Results

Having scoliosis can change a patient’s life, both positively and negatively. Bracing is a physical, but also mental, challenge, especially in children.

## Conclusion

Scoliosis is a struggle, and not only a temporary problem. Scoliosis is an ongoing conflict. Scoliosis is hard for both patients, and physicians, to fully comprehend. Patients need time to adjust, and come to good terms with their situation. Patients must be able to understand what scoliosis is, the different treatment options they have, and how scoliosis will affect their lives in many ways as well. Physicians must also learn how to speak to their patients, in unbiased terms towards different methods of scoliosis treatment, and additionally, come to acknowledge that patients are humans that can feel pain, and are emotionally affected by scoliosis.

